# Miscellanies

**Published:** 1821-03-01

**Authors:** 


### MISCELLANIES.

SOME ATHENIANS of the profession have observed that the pages of the Medico-Chirurgical Review are not sufficiently pregnant with attic salt and *criticism*. We have again to state, that we do not spend our midnight hours by the lamp, to gratify the fastidious palates of critics, but to diffuse useful information among those who are actually and zealously employed in the practice of their profession. Neither are we so utopian as to expect to please every class of readers. We have good reason to know that our plan gives satisfaction to the great mass of medical society, and as for the critical tribe,* we are extremely indifferent whether or not they honour us with their notice. What is criticism after all, but the opposition of one man's opinion or sentiments to those of another?—the judgment of an anonymous, *versus* that of a known writer? Whenever criticism, especially in scientific matters, runs beyond plain and liberal commentary, it is too often founded in self-conceit, prejudice, or unworthy feeling.

Mature reflection and some experience have convinced us, that the surest way of doing justice to an author is, to enable the *Public* to form their judgment from an impartial analysis of his work. In *this* case the judgment is always just—when the reviewer takes the decision entirely into his own hands, he is not seldom wrong. To the Public at large, the advantages of analysis over criticism are beyond all comparison. In the former case they have *things* set before them—in the latter, *opinions*. We know pretty well which they prefer, and we shall act accordingly.

---

*Obituary.*

Dr. DICKSON of Clifton, has lately announced the death of Mr. BAYNTON of Bristol, and paid to the memory of his friend the last tribute which friendship can bestow—"*in cineres cum corpora jacent.*" To those around him, he was well known for the goodness of his heart, and the kindness of his disposition. "Sanguine in prognosis, and successful in practice, he was equally fortunate in gaining the confidence, and in preserving the friendship, of his patients, by a large circle of whom, he is deeply and deservedly regretted." It is in vain to deplore the inevitable stroke of that sword which—"florem roboris, et seræ spem præsidiumque senectæ prostravit." The best incense we can offer at the shrines of the dead, is the imitation of their virtues.

---

*Experiments on Hydrophobia.* By M. Magendie, M.D.

M. Magendie remarks that, both in animals and men labouring under Hydrophobia, the most active substances, the most powerful narcotics, have no perceptible operation. This holds good, not only as to matters taken into the stomach, but injected into the veins. For instance, he has injected into the veins of dogs that were hydrophobic large doses of opium, (ten grains) without any perceptible narcotic effect; while a single grain produced eight or ten hours of somnolency in a healthy animal of the same species. It was the same in man. M. Dupuytren and our author injected into the radial vein of a young man, in rabies canina, about eight grains of the gummy extract of opium, without any apparent result. Prussic acid was also injected into the vessels of dogs, with the same want of effect. M. M. Magendie and Breschet inoculated a healthy dog with the saliva of the young man abovementioned, by inserting some of the fluid under the skin of the forehead. The animal became mad at the end of a month. Two dogs, bitten by the latter, became affected with hydrophobia in forty days. These last bit several other dogs, but without effect. So that, according to these experiments, the virus becomes innocuous in the third inoculation or generation. But to come to the main experiment of this paper.

The proprietor of a kind of menagerie, in Paris *(le combat des animaux)*, sent for M. Magendie to see a very large and strong bitch, in a high state of rabies. The constant agitation of the animal—hoarse and short barkings—and fierce expression of the eye, convinced our author that the animal was hydrophobic. Early next morning, M. Magendie, attended by several of his most zealous pupils, secured the animal, with some difficuty and hazard. M. Magendie then opened the left jugular vein, and drew off about sixteen ounces of blood; after which, he injected nearly forty ounces of water, during the latter part of the operation, however, permitting ten or twelve ounces of blood and water to flow from the upper part of the orifice. The injection finished, the dog was let loose into her den; and to their great surprize, coiled herself up and lay down, as if to sleep, in the most perfect state of calmness. The fierce expression of the eye was entirely gone—she did not bark, and only ground the teeth, when a stick was put into her cage. M. Magendie waited an hour, during which the animal lay perfectly quiet. Some pupils were left to watch her. About five hours afterwards, she was seized with a difficulty of breathing, which increased, and killed her in half an hour more. On dissection the brain, spinal marrow, and all the organs, excepting the lungs were sound. The lungs were gorged with watery blood, and the mucous membrane appeared inflamed. M. Magendie, before the fatal termination of this case, accused himself of having injected too much water, and anticipated effusion in the lungs. What led him to this experiment? It was from observing that, in artificial aqueous plethora, the various functions of the animal, especially those of the nervous system, were very evidently enfeebled. Now, in rabies, the excitement of the nervous system is carried to its utmost limit; and hence he was naturally enough led to try the sedative effects of aqueous injection and bleeding. Moreover, from the time an animal becomes mad, he ceases to drink; while the pulmonary and cutaneous transpirations are in full force. Hence he found the blood of rabid animals thick, and apparently without serum. Upon the whole, this experiment, though unsuccessful, holds out a ray of hope in this hitherto incurable affliction.—*Magendie's Journal of Physiology, No. I, January,* 1821.

---

Journal of Experimental Physiology, conducted by Dr. MAGENDIE.

We have received a letter from Dr. MAGENDIE, inclosing the prospectus of a Quarterly Journal of Physiology, founded solely on facts: the first number of which appeared in January, 1821, consisting of about 100 pages. The well-merited reputation which this physician has long enjoyed, as an experimental physiologist, will doubtless insure a favourable reception, in the Medical World, for a periodical work dedicated to so interesting a subject, and one so little advanced, as Physiology. We have taken steps to exchange Journals with Dr. MAGENDIE, and shall embrace the earliest opportunity of giving a more particular account of the work now announced to the British Public. *See preceeding article.*

